# Utilization of a Machine Learning Algorithm for the Application of Ancillary Features to LI-RADS Categories LR3 and LR4 on Gadoxetate Disodium-Enhanced MRI

**DOI:** 10.3390/cancers15051361

**Published:** 2023-02-21

**Authors:** Seongkeun Park, Jieun Byun, Sook Min Hwang

**Affiliations:** 1 Machine Intelligence Laboratory, Department of Smart Automobile, Soonchunhyang University, Asan 31538, Republic of Korea; 2 Department of Radiology, College of Medicine, Ewha Womans University, Seoul 07804, Republic of Korea; 3 Department of Radiology, Hallym University College of Medicine, Kangnam Sacred Heart Hospital, Seoul 07441, Republic of Korea

**Keywords:** hepatoceullular carcinoma, Liver Imaging Reporting and Data System, MRI

## Abstract

**Simple Summary:**

In the Liver Imaging Reporting and Data System (LI-RADS), liver observations are categorized as LR1-LR5 according to the probability of benign and hepatoma on the basis of major features. Subsequent adjustment is allowed using ancillary features (AFs). However, the LI-RADS does not provide specific guidelines. In this study, we determined the utilization of a machine-learning-based strategy of applying AFs to LR3/4 on MRI. Our decision tree algorithm of applying AFs for LR3/4 provides significantly higher AUC, sensitivity, and accuracy than those of other methods, albeit reduced specificity. These appear to be usefully employed in certain circumstances in which there is a focus on the early detection of hepatoma.

**Abstract:**

Background: This study aimed to identify the important ancillary features (AFs) and determine the utilization of a machine-learning-based strategy for applying AFs for LI-RADS LR3/4 observations on gadoxetate disodium-enhanced MRI. Methods: We retrospectively analyzed MRI features of LR3/4 determined with only major features. Uni- and multivariate analyses and random forest analysis were performed to identify AFs associated with HCC. A decision tree algorithm of applying AFs for LR3/4 was compared with other alternative strategies using McNemar’s test. Results: We evaluated 246 observations from 165 patients. In multivariate analysis, restricted diffusion and mild–moderate T2 hyperintensity showed independent associations with HCC (odds ratios: 12.4 [*p* < 0.001] and 2.5 [*p* = 0.02]). In random forest analysis, restricted diffusion is the most important feature for HCC. Our decision tree algorithm showed higher AUC, sensitivity, and accuracy (0.84, 92.0%, and 84.5%) than the criteria of usage of restricted diffusion (0.78, 64.5%, and 76.4%; all *p* < 0.05); however, our decision tree algorithm showed lower specificity than the criterion of usage of restricted diffusion (71.1% vs. 91.3%; *p* < 0.001). Conclusion: Our decision tree algorithm of applying AFs for LR3/4 shows significantly increased AUC, sensitivity, and accuracy but reduced specificity. These appear to be more appropriate in certain circumstances in which there is an emphasis on the early detection of HCC.

## 1. Introduction

The Liver Imaging Reporting and Data System (LI-RADS) was released in 2011 by the American College of Radiology [1] and was continuously updated until 2018 to improve diagnostic accuracy and promote communication between healthcare providers by standardizing the interpretation and categorization of liver observations. In the LI-RADS, liver observations are categorized as LR1 to LR5 according to the probability of benignity and hepatocellular carcinoma (HCC), though the algorithm includes the size and major features (MFs) such as nonrim arterial phase hyperenhancement (APHE), enhancing capsule, nonperipheral washout, and threshold growth. After allocation to a category based on the MFs, adjustment using ancillary features (AFs) is allowed [2]. Observations can be upgraded by one category up to LR4 using AFs favoring malignancy, whereas they can be downgraded by one category using AFs favoring benignity. Based on a recent meta-analysis, the occurrence rate of HCC is 0% in LR1, 13% in LR2, 38% in LR3, 74% in LR4, and 94% in LR5 [3], although these rates in the lower categories may be inflated owing to selection bias for biopsied lesions. Once the LR category is assigned to each observation, cases with LR3 and LR4 observations are recommended for repeat or alternative diagnostic imaging in 3–6 months, multidisciplinary discussion, or biopsy. However, an invasive biopsy is a risky procedure and may result in biopsy failure because small lesions are classified as mainly LR3 or LR4. In addition, there is a risk of missing local treatment opportunities or a decrease in the number of treatment options due to increased lesion size or vascular invasion during follow-up without treatment. The LI-RADS does not provide specific guidelines for the application of Afs, and the utilization of AFs are at the discretion of the radiologist according to each case. Furthermore, variability of the proportion of the change in the LI-RADS category has been shown after the application of AFs among the studies, that is, from 18.1% to 56.4% [4,5,6]. Studies have shown that some observations remained in the initial category even after the application of AFs; therefore, specific and appropriate instructions for the application of AFs are necessary to improve the accuracy and timeliness of diagnosis. Prior studies have reported the widely variable performance of AFs of 3~62% in sensitivity and 79~99% in specificity [5,7,8,9], and certain AFs, such as mild–moderate T2 hyperintensity or hepatobiliary hypointensity, showed stronger associations than other AFs. However, some studies were limited to the analysis of hepatic observations already categorized as LR5; thus, these studies involved unavoidable inflated sensitivity and unreliable significant association of AFs for diagnosing HCC. Those studies reported various rules for applying AFs for improving the diagnostic performance of LR3 and LR4 observations on gadoxetate disodium-enhanced MRI (using 2–4 or more AFs or specific combinations using independent features identified by multivariate analysis) [4,10,11,12].

Recently, artificial intelligence has been actively utilized in many complex problems to facilitate the identification of complex patterns and relationships within various parameters. It has the potential to rapidly evolve into an applicable solution in the medical field to improve diagnostic accuracy, treatment strategy, and follow-up outcomes [13,14]. However, to our knowledge, no study has examined the effect of machine learning algorithms on applying AFs in the LI-RADS.

Therefore, this study aimed to identify the important features of AFs and determine the utilization of a machine-learning-based strategy for applying AFs to LR3 and LR4 observations on gadoxetate disodium-enhanced MRI.

## 2. Materials and Methods

This retrospective study was conducted at a single center after approval was obtained from the institutional review board. The requirement for informed consent was waived due to the retrospective nature of the study.

Interventional studies involving animals or humans and other studies that require ethical approval must list the authority that provided approval and the corresponding ethical approval code.

### 2.1. Study Subjects

We searched our institution’s electronic medical records and identified 523 treatment-naïve patients at risk for HCC who underwent gadoxetate disodium-enhanced MRI between January 2017 and February 2022. We included patients who met the following criteria: (1) age ≥ 18 years; (2) high risk for HCC according to the LI-RADS v2018 (presence of cirrhosis or chronic hepatitis B infection regardless of the presence of cirrhosis); and (3) MRI findings of a focal hepatic solid nodule. We excluded 356 patients based on the following criteria: (1) inadequate final diagnosis such as unknown final diagnosis of malignancy as a result of immediate locoregional therapy or insufficient follow-up (<2 years) for benign lesions to determine size stability (*n* = 308); (2) poor quality of images for interpretation (*n* = 3); (3) only observations categorized as LR1, LR2, LR5, LR-TIV, or LR-M, according to the LI-RADS v2018 MFs and the algorithm (*n* = 45). Finally, 167 patients (132 males and 35 females; mean age, 62.8 ± 8.4 years) in whom gadoxetate disodium-enhanced MRI showed at least one untreated LR3 or LR4 observation were included in this study as a development group. For external validation, we collected 25 temporally separated patients who underwent gadoxetate disodium-enhanced MRI between March 2022 and October 2022. Excluding 5 patients due to nonavailable final diagnosis, 20 patients (17 males and 3 females; mean age, 64.4 ± 10.6 years) were enrolled as a test group. The LR3 and LR4 observations were categorized using only the MFs per the LI-RADS v2018 as follows: LR3, nonrim APHE, size 10–19 mm, and no additional MFs; LR4, nonrim APHE, size 10–19 mm, and enhancing “capsule” only as MFs; or nonrim APHE, size ≥ 20 mm, and no additional MFs. We did not consider threshold growth because we included only observations that first presented on gadoxetate disodium-enhanced MRI.

### 2.2. MRI Techniques

The gadoxetate disodium-enhanced liver MRI examinations were conducted using a 3-T MRI scanner (MAGMETOM Vida, Siemens Healthcare; SIGNA Architect, GE, Erlangen, Germany). The imaging protocol included the following sequences: axial T2-weighted single-shot fast spin echo; axial T2-weighted fast spin echo; axial dual-gradient-recalled echo (GRE) T1-weighted sequence (in-phase and opposed-phase); and axial T1-weighted three-dimensional (3D) GRE with fat suppression (liver acquisition with volume acceleration—LAVA, or volumetric interpolated breath-hold examination—VIBE) obtained before and after the intravenous bolus injection of 0.025 mmol/kg gadoxetate disodium at a rate of 1.0 mL/s, followed by a subsequent 20 mL saline flush. Postcontrast axial 3D GRE images were obtained during the late hepatic arterial phase (AP; 5 s after peak aortic enhancement determined using 1 mL test bolus injection), portal venous phase (PVP; 50 s), transitional phase (TP; 3 min), and hepatobiliary phase (HBP; 20 min). Diffusion-weighted images were acquired using a maximum b-value of 800 s/mm^2^. Details of the MRI parameters are shown in Table A1 in Appendix A.

### 2.3. Image Analysis

Image analyses were performed by two board-certified radiologists with >9 years of experience in hepatic imaging, who were blinded to any information about clinical history or final diagnosis. Any disagreement was resolved in consensus. Nodule size and the presence or absence of MFs (nonrim APHE, nonperipheral washout, or enhancing capsule) according to the LI-RADS v2018 were analyzed. The following AFs were also assessed based on the LI-RADS v 2018: (1) AFs favoring malignancy in general: mild–moderate T2 hyperintensity, corona enhancement, fat sparing in a solid mass, iron sparing in a solid mass, TP hypointensity, HBP hypointensity, and restricted diffusion; and (2) AFs favoring malignancy in particular: nonenhancing capsule, nodule-in-nodule, mosaic architecture, blood products in mass, and fat in mass more than that in the adjacent liver. Imaging features regarding interval change in tumor size (threshold growth, subthreshold growth, size stability ≥ 2 years, or size reduction) and discrete nodules observed on ultrasound were not assessed because only focal lesions initially detected on MRI were included, and prior imaging studies for the comparison were not provided. AFs favoring benignity were also not analyzed in this study because no observation showed AFs favoring benignity in preliminary imaging analysis.

### 2.4. Reference Standard

The diagnosis of HCC was performed through histopathological confirmation or diagnosis of definitive HCC (LR5) on follow-up imaging (either contrast-enhanced computed tomography [CT] or MRI) within 12 months [5,11,12,15,16]. Benignity was diagnosed based on histopathological results or shrinkage or stability of the solid mass as seen on follow-up serial imaging (contrast-enhanced CT or MRI) for a minimum of 24 months [5,11,12,15,16,17]. The mean imaging follow-up period for benign observations was 28.1 ± 23.0 (range, 24.7–52.3) months. Histopathological diagnosis was performed after surgical resection (*n* = 11) or core-needle biopsy (*n* = 30).

### 2.5. Extracting Important Features and Constructing a Machine-Learning-Based Algorithm for Applying AFs

Evaluating the influence of each factor is important for understanding the prediction process. In this study, we evaluated the feature importance using a random forest model. A random forest [18] is an ensemble of decision trees. First, we sampled a dataset by allowing duplication from the training data, and many decision trees were generated using the sampled data. Second, various types of tree structures, which are called clusters of random forest trees, were generated. Important features are located at the top of each tree to predict the results, and the importance of each feature can be determined by statistically analyzing the number of trees.

After determining the feature importance by random forest, we used the decision tree model for HCC prediction. A decision tree [18] is one of the most famous machine learning algorithms. The advantage of a decision tree is that it is very intuitive and explainable for classification and regression; therefore, it is easy to understand why the results are predicted by the decision tree. In other words, unlike other machine learning algorithms such as KNN and SVM, a decision tree has the advantage of using both the results and a prediction process because of its explainability. In this study, we used scikit-learn (v1.1.1) [19] in Python for decision tree training. The classification and regression tree (CART) method in scikit-learn is used to train the decision tree. The CART divides the data into two subsets depending on the characteristics that distinguish the data in the training set. The CART sets a threshold for one input feature, divides the data into two subsets according to the threshold, and sets the input factor and its threshold to minimize the impurity between the two divided subsets. The cost function of the classification CART is described by the following Equation (1):

(1) $$J\left(k,t_{k}\right)=\frac{m_{left}}{m}G_{left}+\frac{m_{right}}{m}G_{right}$$

where is the kth feature of the input, $t_{k}$ is the threshold of the kth feature of the input, $G_{left/right}$ is the impurity of the left/right subset, $m_{left/right}$ is the number of samples of the left/right subset, and m is the total number of samples. The CART builds its subtree using a recursive method of dividing the subset. The threshold is determined using two criteria: GINI and entropy. In this study, we used the GINI method to determine the threshold of the subset tree.

Most ML algorithms cannot be reproduced due to the random characteristics of some hyperparameters. To solve the random characteristics of these ML algorithms, most ML libraries, including scikit learn, fix the random characteristics, enabling the generation of reproducible random variables. In other words, we used the randomness fixing technique of scikit-learn to perform fixing of both the randomnesses of the data splits and the hyperparameters.

### 2.6. Statistical Analysis

All statistical analyses were performed on an observational basis. Continuous variables are presented as mean ± standard deviation (SD) or as median and interquartile range (IQR) and compared between HCC and non-malignant nodules using the Student’s t-test or the nonparametric Mann–Whitney U test. Categorical variables or MFs and AFs are expressed as numbers and frequencies and compared using chi-squared test or Fisher’s exact test, as appropriate. To identify significant AFs suggestive of HCCs rather than non-malignant nodules in initial LR3 or LR4 observations (LR3 or LR4 determined with only MFs, regardless of AFs), univariate and multivariate logistic regression analyses were performed. In the multivariate analysis, variables that showed a positive association with HCC (*p* < 0.05) in the univariate analysis were entered, and backward stepwise elimination was performed. The inter-reader agreement was evaluated using kappa statistics.

The important AFs were identified using random forest analysis, and a decision tree algorithm was constructed for the application of AFs to improve diagnostic performance in the LR3 and LR4 categories.

Sensitivity, specificity, accuracy, and area under the receiver operating characteristic (ROC) curve (AUC), positive predictive value, and negative predictive value were calculated to evaluate the diagnostic performance of our diagnostic systems. Statistical significance was set at *p* < 0.05. Statistical analyses were performed using SPSS version 25.0 (IBM Inc., Armonk, NY, USA) and MedCalc version 19.4.0 (MedCalc Software). The important AFs were identified, and a decision tree algorithm for applying AFs to the LR3 and LR4 categories was constructed using Python 3.8.13 module scikit-learn (v1.1.1) (Python Software Foundation, Wilmington, DE, USA).

## 3. Results

### 3.1. Baseline Characteristics of Patients and Observations

The final study sample included 167 patients (mean age, 62.8 years; range, 33–84 years) with 245 observations (median size, 13 mm; IQR 10–17.8 mm) in the development group and 20 patients (mean age, 64.4 years; range, 37–81 years) with 30 observations (median size, 11 mm; IQR 6–23 mm) (Table 1) in the test group. Among 167 patients, hepatitis B was the most common cause of liver cirrhosis or chronic liver disease (*n* = 117, 77%). One hundred twelve (67.1%) patients had one lesion, 36 (23.7%) had two lesions, and 19 (12.5%) had three or more lesions. The median size of 245 observations was 13 mm (IQR, 10–17.8 mm). Among MFs, nonrim APHE was observed in 96 (39.2%) nodules, nonperipheral washout in 74 (30.2%) nodules, and enhancing capsule in 15 (6.1%) nodules. Overall, 137 HCC lesions and 108 benign lesions were identified. The test group included 20 patients.

### 3.2. Comparison of Imaging Features between HCCs and Non-Malignant Nodules and Important Features for Diagnosis HCC in LR3 and LR4 Observation

**Figure 1:**
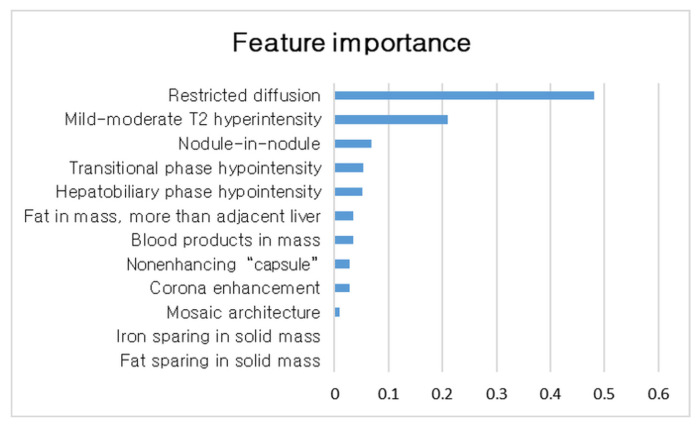
Ranking of feature importance.

Comparative analyses of the imaging features between HCC and non-malignant nodules are summarized in Table 2. The most common AFs recorded in HCC were HBP hypointensity (131, 95.6%), followed by TP hypointensity (113, 82.5%), restricted diffusion (88, 64.2%), and mild–moderate T2 hyperintensity (86, 62.8%). Fat sparing and iron sparing in the solid mass were not observed. Univariate analyses demonstrated that restricted diffusion, mild–moderate T2 hyperintensity, TP hypointensity, HBP hypointensity, nonenhancing capsule, and nodule-in-nodule appearance were significantly associated with HCC (all *p* < 0.05). In the multivariate analyses, restricted diffusion (odds ratio [OR], 12.4; 95% confidence interval [CI], 5.1–30.35; *p* < 0.001) and mild–moderate T2 hyperintensity (OR, 2.5; 95% CI, 1.1–5.3; *p* = 0.02) were independent significant features associated with HCC (Table 3). Random forest analysis showed restricted diffusion as the most important feature (feature importance ratio: 0.48), followed by mild–moderate T2 hyperintensity (feature importance ratio: 0.21; Figure 1). Interobserver agreement of the AFs are presented in Table A2. We observed a range from 0.21 to 0.74 of kappa values for each AF. Among the AFs, hepatobiliary-phase hypointensity showed the highest kappa value. Restricted diffusion and mild–moderate T2 hyperintensity showed moderate agreement with kappa value of 0.55, in both. Nodule-in-nodule architecture showed a relatively high proportion of agreement (82.7%). However, it showed the lowest kappa value (0.21) due to its low prevalence.

### 3.3. Development of Decision Tree Algorithm for Application of AFs to LR3 and LR4 Observation

**Figure 2:**
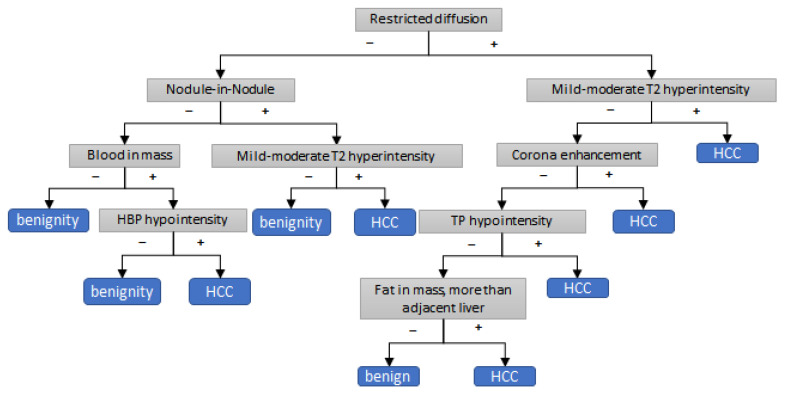
Decision tree algorithm for applying ancillary features to LR3 and LR4 categories.

Figure 2 shows the decision tree algorithm for the application of AFs to LR3 and LR4 observations for HCC diagnosis. Restricted diffusion was the first partitioning imaging feature of the decision tree algorithm. Further branching was performed using other features such as nodule-in-nodule, mild–moderate T2 hyperintensity, blood in mass, TP hypointensity, corona enhancement, HBP hypointensity, and fat in mass. This decision tree algorithm yielded an AUC of 0.84 (95% CI, 0.84–0.85); sensitivity of 92.0% (95% CI, 91.6–92.4); specificity of 71.1% (95% CI, 70.9–71.4); and accuracy of 84.5% (95% CI, 84.1–84.8) in the development group. In the test group, the decision tree algorithm showed good diagnostic performance: an AUC of 0.82 (95% CI, 0.76–0.88); sensitivity of 94.0% (95% CI, 89.3–98.2); specificity of 68.7% (95% CI, 58.0–79.4); and accuracy of 81.9% (95% CI, 75.8–87.9).

### 3.4. Comparison of Diagnostic Performance of Decision Tree Algorithm with Alternative Criteria of Applying Afs

**Figure 3:**
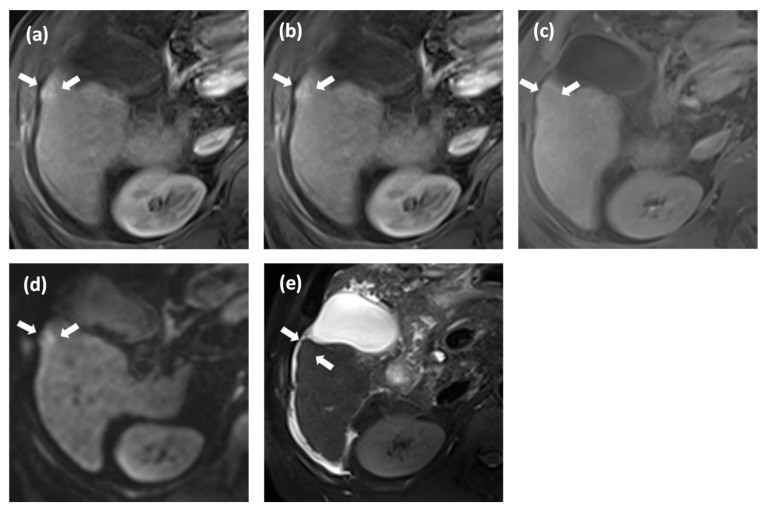
Histologically proven hepatocellular carcinoma (HCC) in a 67-year-old man with hepati-tis-B-virus-related liver cirrhosis. Gadoxetate disodium-enhanced MRI (**a**–**e**, arrow) shows a 10-mm observation with nonrim arterial phase hyperenhancement (**a**) and lack of definitive nonperipheral washout or enhancing capsule on portal venous (**b**) and transitional phase image (**c**). Thus, it is categorized as LR3 according to major features only. The observation shows restricted diffusion (b = 800 s/mm^2^; (**d**)) and mild T2 hyperintensity (**e**). Our decision tree algorithm classifies this nodule as HCC.

**Figure 4:**
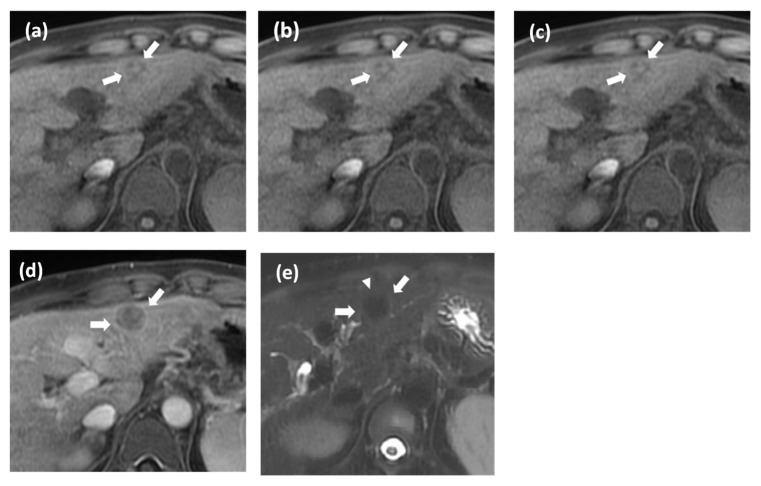
Histologically proven hepatocellular carcinoma (HCC) in a 35-year-old woman with chronic hepatitis B. Gadoxetate disodium-enhanced MRI (**a**–**e**, arrow) shows a 17-mm observation with a smaller inner nodule showing different imaging characteristics than the larger outer nodules on precontrast T1 image (**a**). This demonstrates the nodule-in-nodule. This observation does not show nonrim arterial phase hyperenhancement (**b**) in the nonperipheral washout on portal venous phase. (**c**) It shows enhancing capsule in the portal venous (**c**) and transitional phase (**d**). Thus, it is cate-gorized as LR4 according to major features only. The observation shows suspicious mild T2 hy-perintense foci within the nodule (arrowhead; (**e**)). Our decision tree algorithm classifies this nodule as HCC.

**Figure A1:**
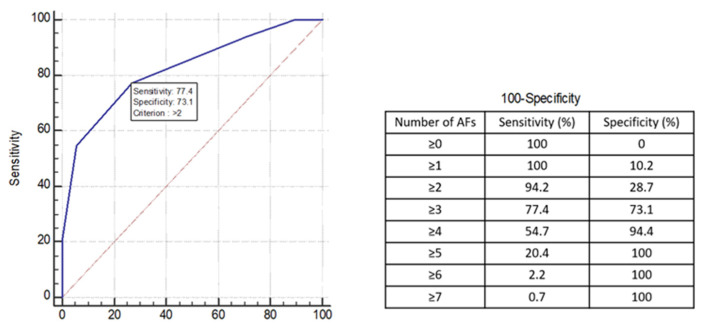
Receiver operating characteristic curve of the number of ancillary features favoring malignancy for the diagnosis of hepatocellular carcinoma and diagnostic performance of each criterion values.

We also established alternative criteria for the application of Afs to LR3 and LR4 for HCC diagnosis. The diagnostic performance of these criteria for the application of Afs (according to the number of Afs and exclusive usage of significant Afs or their combination) is presented in Table 4. Figure A1 in Appendix A shows the diagnostic performance of the application criteria according to the number of Afs favoring malignancy at every cutoff point, that is, the number of Afs ≥ 1 to ≥6. A cutoff value of ≥3 showed the highest diagnostic performance, with an AUC of 0.75 (95% CI, 0.75–0.76); sensitivity of 77.6% (95% CI, 76.4–78.8); specificity of 72.9% (95% CI, 72.2–73.7); and accuracy of 75.5% (95% CI, 75.0–76.1). We also analyzed the application criteria via various combinations of independently significant AFs (restricted diffusion and mild–moderate T2 hyperintensity) identified from the multivariate and random forest analyses. Among those criteria, the criterion of “restricted diffusion only” yielded the highest AUC (0.78) compared with the criterion of “restricted diffusion or mild-moderate T2 hyperintensity” (AUC, 0.76; *p* = 0.025); “restricted diffusion and mild-moderate T2 hyperintensity” (AUC, 0.75; *p* = 0.01); and “mild-moderate T2 hyperintensity only” (AUC, 0.73; *p* < 0.001; Table A3 in Appendix A). Our decision tree approach had higher AUC, sensitivity, and accuracy than the other criteria (all *p* ≤ 0.002, Figure 3 and Figure 4); however, it showed a significantly reduced specificity compared with the criterion of “restricted diffusion only” (Table 4).

## 4. Discussion

The current study revealed two AFs favoring malignancy (restricted diffusion and mild–moderate T2 hyperintensity) as significant independent features for the diagnosis of HCC in LR3 and LR4 observations on gadoxetate disodium-enhanced MRI in both multivariate and random forest analyses. Furthermore, we presented a strategy for applying AFs in the diagnosis of HCC from the initial LR3 and LR4 observations, which are categorized using only the MFs of the LI-RADS v2018. We developed a decision tree algorithm to apply AFs to LR3 and LR4 observations. This approach was compared with other alternative approaches using the number of AFs or various combinations of significant AFs identified from multivariate and random forest analyses. Our decision tree approach showed the highest AUC, sensitivity, and accuracy compared with other criteria, albeit with somewhat compromised specificity.

Our results showed that restricted diffusion and mild–moderate T2 hyperintensity were independent and important features for the diagnosis of HCC from the LR3 and LR4 observations in both multivariate and random forest analyses. In particular, restricted diffusion showed the highest odds ratio (12.4; 95% CI, 5.1–30.0) and importance ratio (0.48), which is consistent with the results of previous studies [20,21,22]. Diffusion restriction is not specific to HCC but is more often used for detecting hepatic lesions and discriminating malignant lesions from benign lesions [23,24]. It is important to understand hepatocarcinogenesis in the early diagnosis of HCC from a premalignant lesion such as a dysplastic nodule. Hepatocarcinogenesis is a multi-step process that starts from a regenerative nodule in cirrhosis or as a dysplastic nodule and progresses to advanced HCC [25]. Given that one of the major histologic differences between dysplastic nodules and early HCC is the degree of cellular density, restricted diffusion reflecting the high cellularity of lesions might help in the better discrimination of HCC from non-malignant lesions [15].

T2 hyperintensity is a typical imaging feature of HCC and helps differentiate hypovascular HCC from dysplastic nodules [26]. We observed that mild–moderate T2 hyperintensity has an OR of 2.5 and an importance ratio of 0.21 and is the second most significant feature after restricted diffusion. According to previous studies, mild–moderate T2 hyperintensity has been proven to be a suggestive feature of progressed HCC rather than early HCC [27,28]. When a focus of HCC develops within a dysplastic nodule, a mildly elevated signal may be observed on T2-weighted images, representing the focus of HCC within the hypointense dysplastic nodule, and has been described as a “nodule-in-nodule” appearance. This is consistent with our results showing that presentations of a nodule-in-nodule appearance were significantly more frequently encountered with HCCs than with non-malignant nodules, although their numbers were small. Thus, the entire change in T2 signal intensity of the observation may reflect the progressive biological characteristics of HCC.

In our study, among AFs favoring malignancy, the most commonly encountered feature was HBP hypointensity. However, this feature was not significantly associated with HCC in the multivariate analysis. This might be because this feature appears to be frequent, even in non-malignant nodules, which is consistent with the results of previous studies [11,29]. Because organic anion-transporting polypeptides that mediate hepatic uptake of gadoxetic acid may decrease in expression in the early stage of hepatocarcinogenesis, dysplastic nodules or regenerative nodules, and even hemangioma cysts, can present with HBP hypointensity [30,31]. Therefore, applying this characteristic to the LR3 and LR4 categories may cause concerns regarding false positivity when diagnosing HCC. In this study, we found consistent results: HBP hypointensity was a significantly frequent finding among AFs in misclassification cases using our decision tree algorithm (Table A4).

In the decision tree algorithm for the application of AFs in LR3 and LR4 observation, the results showed a good ability of the method to diagnose HCC, with an AUC of 0.84 (95% CI, 0.84–0.85); sensitivity of 92.0% (95% CI, 91.6–92.4); specificity of 71.1% (95% CI, 70.9–71.4); and accuracy of 84.5% (95% CI, 84.1–84.8). The decision tree algorithm consists of restricted diffusion, nodule-in-nodule, mild–moderate T2 hyperintensity, blood in mass, TP hypointensity, corona enhancement, HBP hypointensity, and fat in mass. Interestingly, in the decision tree algorithm, nodule-in-nodule, blood in mass, TP hypointensity, corona enhancement, HBP hypointensity, and fat in mass, which failed to demonstrate independent associations in the present study, were correlated with restricted diffusion and mild–moderate T2 hyperintensity. This may indicate that minor AFs still play important roles in the diagnosis of HCC among observations that already exhibit significant weighting features.

We also evaluated an alternative application algorithm using a criterion based on the number of AFs and criterion utilizing independent features identified by multivariate analysis. These approaches have been addressed in previous studies [10,11,12,32]. Kang et al. reported that criteria with the number of AFs ≥ 4 showed a sensitivity of 80.6% and a specificity of 70.0% [12], and Cannella et al. reported that criteria with the number of AFs ≥ 2 showed a sensitivity of 72.6% and a specificity of 91.5% [11]. The present study also showed good diagnostic ability for HCC using criteria with the number AFs ≥ 3, with a sensitivity of 77.6% and a specificity of 72.9%. Direct comparison between results among the studies may be unnecessary because of the differences in the characteristics between study populations. Nevertheless, our results have strength in overcoming the overestimation of sensitivity because of the exclusion of the LR5 observation. In addition, this approach showed significantly lower sensitivity and specificity than those of the decision tree algorithm.

Cannella et al., Lee et al., and Jeon et al. showed how to incorporate AFs identified as significant independent features in multivariate analysis to enhance diagnostic performance in the LR3 and LR4 categories in the LI-RADS diagnostic table [10,11,32]. In the present study, we identified that the highest diagnostic performance for HCC was achieved using the exclusive application of restricted diffusion to the LR3 and LR4 categories, among other combinations of significant AFs. This approach had significantly higher specificity than our decision tree algorithm (91.3% vs. 71.1%, *p* < 0.001). Nevertheless, our decision tree algorithm showed significantly higher AUC, sensitivity, and accuracy in HCC diagnosis in LR3 and LR4 observations (AUC, 0.84 vs. 0.78, *p* < 0.001; sensitivity, 92.0% vs. 64.5%, *p* < 0.001; and accuracy, 84.5% vs. 76.4%, *p* = 0.032) than that of the criteria utilizing the exclusive application of restricted diffusion to the LR3 and LR4 category. Indeed, the LI-RADS is designed to promote the specific diagnosis of HCC [1]. Other Western countries also adopt specific diagnostic algorithms to avoid false-positive diagnoses of HCC, because liver transplantation is the only potentially curative treatment in patients with advanced cirrhosis, who predominantly constitute individuals with a high risk of HCC in Western countries [33,34]. Meanwhile, Asian countries prefer sensitive diagnosis of HCC to detect HCC in its early stages and to provide patients with HCC with local treatment, such as resection or ablation, as a curative treatment [33,34]. Therefore, despite significantly reduced specificity, our decision tree algorithm for the application of AFs with significantly high sensitivity can be used more in Asian societies.

This decision tree algorithm is a conceptually simple decision-making model and provides the diagnosis process of HCC in an easy-to-understand classification system. Thus, it may be useful in situations in which a decision must be made effectively and reliably. Although there were some limitations in the absence of AFs favoring benignity in our decision tree algorithm, it may be still useful in daily practice, as compared with other alternative approach.

Our study had several limitations. First, there may have been an inevitable selection bias owing to the retrospective nature of this study. Among the initially eligible patients, approximately 308 patients were excluded from the study population due to a lack of a final diagnosis. Among these, the majority showed LR4 observation. They tended to be treated with locoregional treatment without pathological confirmation, especially when they exhibited a co-existing LR5 observation. Second, our study conducted LR3 and LR4 observations simultaneously. As LR3 and LR4 may express different distributions of AFs between HCC and non-malignant nodules, subgroup analysis of LR3 and LR4 showed more confident study results. Although subgroup analysis could not be performed in this study due to the lack of LR4 lesions, a larger study should be conducted in the future. Lastly, the majority of benign lesions in this study were not confirmed using biopsy but by follow-up imaging. To minimize misdiagnosis, we considered benignity based on long-term stability (≥24 months), whereas HCC was considered based on the presence of LR5 observation on follow-up imaging [5,11,12,16,17,26].

## 5. Conclusions

In conclusion, among the LI-RADS v2018 AFs favoring malignancy, restricted diffusion and mild–moderate T2 hyperintensity showed a strong association for the diagnosis of HCC in LR3 and LR4 observations. Our decision tree algorithm for applying AFs to LR3 and LR4 observations provides significantly increased AUC, sensitivity, and accuracy but reduced specificity. These appear to be more appropriate for application under certain circumstances with an emphasis on early detection of HCC.

## Figures and Tables

**Table 1:** Baseline characteristic of 245 observations in 167-patient development group and 30 observations in 20-patient test group.

Characteristics		Development Group	Test Group
Age *	62.8 ± 8.4		64.4 ± 10.6
Sex	Male	132 (79.0)	17 (85.0)
	Female	35 (23.)	3 (15.0)
Underlying disease	Hepatitis B with/without cirrhosis	117 (77.0)	13 (65.0)
	Hepatitis C with cirrhosis	10 (6.6)	2 (10.0)
	Alcoholic liver disease with cirrhosis	30 (19.7)	4 (20.0)
	NASH with cirrhosis	3 (2.0)	0 (0.0)
	Cryptogenic cirrhosis	7 (4.6)	1 (5.0)
Laboratory ^†^	AST (IU/L)	36 (27.0, 52.5)	46 (25, 96)
	ALT (IU/L)	28 (19.0, 41.8)	28 (6, 148)
	Total bilirubin (mg/dL)	0.8 (0.5, 1.2)	1.2 (0.4, 5.7)
	Prothrombin time (s)	12.7 (12.0, 13.6)	13.3 (12, 22)
	Platelet (×1000/μL)	123 (82.5, 177.5)	116 (37, 299)
	AFP (ng/mL)	7.1 (3.5, 26.7)	10.4 (1.8, 239)
No. of nodules included in the analysis	1	112 (73.7)	13 (65.0)
	2	36 (23.7)	4 (20.0)
	3	16 (10.5)	3 (15.0)
	4	2 (1.3)	
	5	1 (0.7)	
LiRADS category	LR3	199 (81.2)	20 (66.7)
	LR4	46 (18.8)	10 (33.3)
Major feature	Size ^†^	13 (10.0, 17.8)	11 (6, 23)
	APHE	96 (39.2)	20 (66.7)
	Non-peripheral washout	74 (30.2)	9 (30.0)
	Enhancing capsule	15 (6.1)	6 (20.0)
Reference standard	Pathologic diagnosis	41 (16.7)	11 (36.7)
	Imaging follow-up	204 (83.3)	19 (63.3)
Final diagnosis	HCC	137 (55.9)	16 (53.3)
	Non-malignant nodule	108 (44.1)	14 (46.7)

Unless otherwise indicated, data are the number of patients with percentages in parentheses. * Data are mean ± standard deviation. ^†^ Data are median value, and data in parentheses are range. NASH—non-alcoholic steatohepatitis; AFP—alpha-fetoprotein; ALT—alanine aminotransferase; AST—aspartate aminotransferase; APHE—arterial phase hyperenhancement.

**Table 2:** Distribution of ancillary features favoring malignancy between HCC and non-malignant nodule.

	Development Group			Test Group		
	HCC (*n* = 137)	Non-Malignant Nodules (*n* = 108)	*p*-Value	HCC (*n* = 16)	Non-Malignant Nodules (*n* = 14)	*p*-Value
**Favoring malignancy in general**						
Corona enhancement	3 (2.2)	1 (0.9)	0.633	1 (6.3)	0 (0.0)	1.0
Fat sparing in solid mass	0 (0.0)	0 (0.0)	NA	0 (0.0)	0 (0.0)	NA
Restricted diffusion	88 (64.2)	9 (8.3)	<0.001	13 (81.3)	0 (0)	<0.001
Mild–moderate T2 hyperintensity	86 (62.8)	19 (17.6)	<0.001	14 (87.5)	1 (7.1)	<0.001
Iron sparing in solid mass	0 (0.0)	0 (0.0)	NA	0 (0.0)	0 (0.0)	NA
Transitional phase hypointensity	113 (82.5)	73 (67.6)	0.007	9 (56.3)	11 (78.6)	0.20
Hepatobiliary phase hypointensity	131 (95.6)	94 (87.0)	0.015	12 (75.0)	12 (85.7)	0.46
**Favoring HCC in particular**						
Nonenhancing “capsule”	11 (8.0)	1 (0.9)	0.011	0 (0.0)	0 (0.0)	NA
Nodule-in-nodule appearance	12 (8.8)	1 (0.9)	0.007	1 (6.3)	0 (0.0)	1.0
Mosaic architecture	5 (3.6)	0 (0.0)	0.069	2 (12.5)	0 (0.0)	0.53
Fat in mass, more than adjacent liver	25 (18.2)	11 (10.2)	0.077	1 (6.3)	3 (21.4)	0.50
Blood products in mass	5 (3.6)	0 (0.0)	0.069	1 (6.3)	0 (0.0)	1.0

Data are the number of patients with percentages in parentheses.

**Table 3:** Univariable and multivariable analysis of AFs associated with HCC.

	Univariable Analysis		Multivariable Analysis	
	OR (95% CI)	*p*-Value	OR (95% CI)	*p*-Value
**Favoring malignancy in general**				
Corona enhancement	2.4 (0.2, 23.4)	0.452		
Fat sparing in solid mass	NA			
Restricted diffusion	19.8 (9.2, 42.5)	<0.001	12.4 (5.1, 30.3)	<0.001
Mild–moderate T2 hyperintensity	7.9 (4.3, 14.5)	<0.001	2.5 (1.1, 5.3)	0.022
Iron sparing in solid mass	NA			
Transitional phase hypointensity	2.3 (1.2, 4.1)	0.008	0.9 (0.4, 2.1)	0.883
Hepatobiliary phase hypointensity	3.3 (1.2, 8.8)	0.020	5.5 (0.9, 32.1)	0.057
**Favoring HCC in particular**				
Nonenhancing “capsule”	9.3 (1.2, 73.5)	0.034	15.8 (0.8, 318.2)	0.072
Nodule-in-nodule appearance	10.3 (1.3, 80.3)	0.026	16.5 (0.9, 142.1)	0.051
Mosaic architecture	1,321,752,144.2 (0.0)	0.999		
Fat in mass, more than adjacent liver	2.0 (0.9, 4.2)	0.081		
Blood products in mass	1,321,752,144.2 (0.0)	0.999		

OR—odds ratio; CI—confidence interval.

**Table 4:** Comparison of various approaches for applying AFs to LR3 and LR4 for diagnosis of HCC.

	AUC (95% CI)	Sensitivity (9% CI)	Specificity (95% CI)	Accuracy (95% CI)	PPV (95% CI)	NPV (95% CI)
I. Decision tree algorithm						
Development cohort	0.84 (0.84, 0.85)	92.0% (91.6, 92.4)	71.1% (70.9, 71.4)	84.5% (84.1, 84.8)	70.5% (70.2, 70.9)	92.2% (91.8, 92.7)
Test cohort	0.82 (0.76–0.88),	94.0% (89.3–98.2)	68.7% (58.0–79.4)	81.9% (75.8–87.9)	70.7% (63.0–78.5)	92.3% (87.5–97.2)
II. Number of AFs ≥ 3	0.75 (0.75, 0.76)	77.6% (76.4, 78.8)	72.9% (72.2, 73.7)	75.5% (75.0, 76.1)	78.3% (77.4, 79.1)	72.2% (70.9, 73.6)
III. Restricted DWI	0.78 (0.77, 0.78)	64.5% (63.6, 65.4)	91.3% (90.8, 91.7)	76.4% (75.7, 77.0)	90.3% (89.9, 90.7)	67.2% (66.1, 68.2)
**Comparison of each approach for applying AFs**						
I vs. II	*p* < 0.001	*p* = 0.002	*p* = 0.886	*p* = 0.017	*p* = 0.216	*p* < 0.001
I vs. III	*p* < 0.001	*p* < 0.001	*p* < 0.001	*p* = 0.032	*p* < 0.001	*p* < 0.001
II vs. III	*p* = 0.011	*p* = 0.024	*p* < 0.001	*p* = 0.899	*p* = 0.025	*p* = 0.469

AUC—area under curve; PPV—positive predictive value; NPV—negative predictive value; CI—confidential interval; AF—ancillary feature; DWI—diffusion-weighted image.

## Data Availability

The data presented in this study are available in this article.

## Appendix A

**Table A1:** Parameters of the MRI sequences.

Sequence								
	T1 LAVA/VIBE		Dual-echo T1 GRE		Navigator-triggered TSE T2		DWI ^†^	
	SIGNA Architect	MAGNETOM Vida	SIGNA Architect	MAGNETOM Vida	SIGNA Architect	MAGNETOM Vida	SIGNA Architect	MAGNETOM Vida
Repetition time (ms)	43.42	3.2	125	164	2100	1300	2600	2300
Echo time (ms)	1	1	1, OP; 3, IP	1, OP; 3, IP	96	83	67	60
Flip angle (°)	10	11	60	57	90	120	90	90
Matrix	260 × 220	352 × 209	260 × 220	320 × 216	260 × 260	256 × 216	260 × 260	130 × 106
Field of view	360 × 360	400 × 338	360 × 360	400 × 338	360 × 360	400 × 338	360 × 360	400 × 326
Section thickness (mm)	4	3	5	5	5	5	5	5
No. of signal acquisitions	1	1	1	1	1	1	4	2

DWI—diffusion-weighted imaging, GRE—gradient-recalled echo, IP—in-phase, NA—not applicable, OP—out-of-phase, T1—T1-weighted, T2—T2-weighted, TSE—turbo-spin echo, VIBE—volumetric interpolated breath-hold examination, LAVA—liver acquisition with volume acceleration. ^†^ Diffusion-weighted imaging was performed using four b-values of 0, 50, 400, and 800 s/mm^2^.

**Table A2:** Inter-observer agreement for AFs in the 274 nodules.

Favoring Malignancy in General, Not HCC in Particular	Agreement Proportion	Kappa Value
Corona enhancement	98.1 (269)	0.66
Restricted diffusion	78.8 (216)	0.55
Mild–moderate T2 hyperintensity	78.8 (216)	0.55
ron sparing in solid mass	100 (274)	0.05
Fat sparing in solid mass	100 (274)	0.50
Transitional-phase hypointensity	85.4 (234)	0.47
Hepatobiliary-phase hypointensity	94.2 (258)	0.74
**Favoring HCC in particular**		
Nonenhancing capsule	96.2 (264)	0.65
Nodule-in-nodule architecture	82.7 (227)	0.21
Mosaic architecture	96.2 (264)	0.48
Fat in mass, more than adjacent liver	92.3 (253)	0.73
Blood products in mass	96.2 (264)	0.49

Data in parentheses are number of nodules. HCC—hepatocellular carcinoma.

**Table A3:** Diagnostic performance of criteria of applying AFs with various combinations of significant associations.

	AUC (95% CI)		Sensitivity (95% CI)				Specificity (95% CI)			Accuracy (95% CI)			PPV (95% CI)			NPV 95% CI)		
(1)	0.78 (0.77, 0.78)		64.5% (63.6, 65.4)				91.3% (90.8, 91.7)			76.4% (75.7, 77.0)			90.3% (89.9, 90.7)			67.2% (66.1, 68.2)		
(2)	0.73 (0.72, 0.73)		63.1% (61.7, 64.5)				82.3% (81.4, 83.1)			71.6% (70.9, 72.3)			81.7% (80.8, 82.6)			64.0% (62.8, 65.1)		
(3)	0.76 (0.75, 0.76)		72.7% (71.8, 73.7)				78.3% (77.5, 79.0)			75.2% (74.8, 75.6)			80.8% (80.0, 81.5)			69.6% (68.5, 70.6)		
(4)	0.75 (0.74, 0.76)		54.9% (53.6, 56.1)				95.3% (94.9, 95.7)			72.8% (71.9, 73.6)			93.6% (93.1, 94.1)			62.7% (61.6, 63.8)		
	**Comparisons of each criterion, *p*-value**																	
	AUC		sensitivity				specificity			accuracy			PPV			NPV		
	(2)	(3)	(4)	(2)	(3)	(4)	(2)	(3)	(4)	(2)	(3)	(4)	(2)	(3)	(4)	(2)	(3)	(4)
(1)	<0.01	0.03	0.01	0.91	0.18	0.13	0.08	0.01	0.37	0.27	0.84	0.42	0.12	0.08	0.6	0.66	0.77	0.48
(2)		<0.01	0.12		0.12	0.209		0.57	0.01		0.42	0.85		0.99	0.03		0.41	0.91
(3)			0.35			0.003			<0.01			0.62			0.02			0.27

(1) Restricted diffusion; (2) mild–moderate T2 hyperintensity; (3) restricted diffusion or mild–moderate T2 hyperintensity; (4) restricted diffusion and mild–moderate T2 hyperintensity; AUC—area under curve; PPV—positive predictive value; NPV—negative predictive value; CI—confidential interval; AF—ancillary feature; DWI—diffusion-weighted image.

**Table A4:** Comparison between true prediction and false prediction using decision tree algorithm in development group.

	True Prediction (*n* = 200)	False Prediction (*n* = 45)	*p*-Value
**Favoring malignancy in general**			
Corona enhancement	3 (1.5)	1 (2.2)	0.56
Fat sparing in solid mass	0 (0.0)	0 (0.0)	
Restricted diffusion	89 (44.5)	8 (17.8)	0.01
Mild–moderate T2 hyperintensity	93 (46.5)	12 (26.7)	0.02
Iron sparing in solid mass	0 (0.0)	0 (0.0)	
Transitional phase hypointensity	154 (77.0)	32 (71.1)	0.4
Hepatobiliary phase hypointensity	180 (90.0)	45 (100.0)	0.03
**Favoring HCC in particular**			
Nonenhancing “capsule”	12 (6.0)	0 (0.0)	0.13
Nodule-in-nodule appearance	12 (6.0)	1 (2.2)	0.472
Mosaic architecture	5 (2.5)	0 (0.0)	0.59
Fat in mass, more than adjacent liver	28 (14.0)	8 (17.8)	0.52
Blood products in mass	5 (2.5)	0 (0.0)	0.59

Data are the number of patients with percentages in parentheses.
